# Traumatic Splenic Infarction and Pulmonary Embolism: A Case Report of Rare Radiological Finding

**DOI:** 10.7759/cureus.12514

**Published:** 2021-01-05

**Authors:** Mithilesh K Sinha, Sudipta Mohakud

**Affiliations:** 1 Surgery, All India Institute of Medical Sciences, Bhubaneswar, IND; 2 Radiology, All India Institute of Medical Sciences, Bhubaneswar, IND

**Keywords:** splenic infarction, splenic injury, traumatic pulmonary embolism

## Abstract

Focused abdominal sonography in trauma (FAST) and contrast-enhanced computed tomography (CECT) abdomen are important radiological tests for evaluating the abdomen in polytrauma cases. When vitals are stable, they help to reach a diagnosis in the majority of patients. However, in a small number of cases they fail to explain the clinical scenario. A continued serial clinical assessment may be helpful in these circumstances. A polytrauma patient was found to be FAST positive. The CT scan revealed pulmonary embolism, splenic infarction, perisplenic and perihepatic hematoma. The patient was complaining of pain abdomen and it worsened on day three of the injury. An exploratory laparotomy was performed. A circumferential intestinal wall hematoma with a tear in mesentery was found. This is a rare case of traumatic splenic infarction with evidence of pulmonary embolism. The serial clinical assessment was helpful as it indicated the need for intervention.

## Introduction

Polytrauma cases with abdominal symptoms are frequent at emergency. Focused abdominal sonography in trauma (FAST) is usually the first investigation in such cases. If FAST is positive and the vitals are stable, then contrast computed tomography scan of the abdomen is performed. But even with this in some cases the diagnosis remains elusive. In presence of persisting symptoms, a serial clinical examination should be performed. Worsening of abdominal symptoms or appearance of abdominal signs should prompt for emergency exploratory laparotomy. In trauma cases with blunt injury to the abdomen, serial clinical examination is very useful especially if there is a suspicion of bowel or mesenteric injury.

## Case presentation

**Figure 1:**
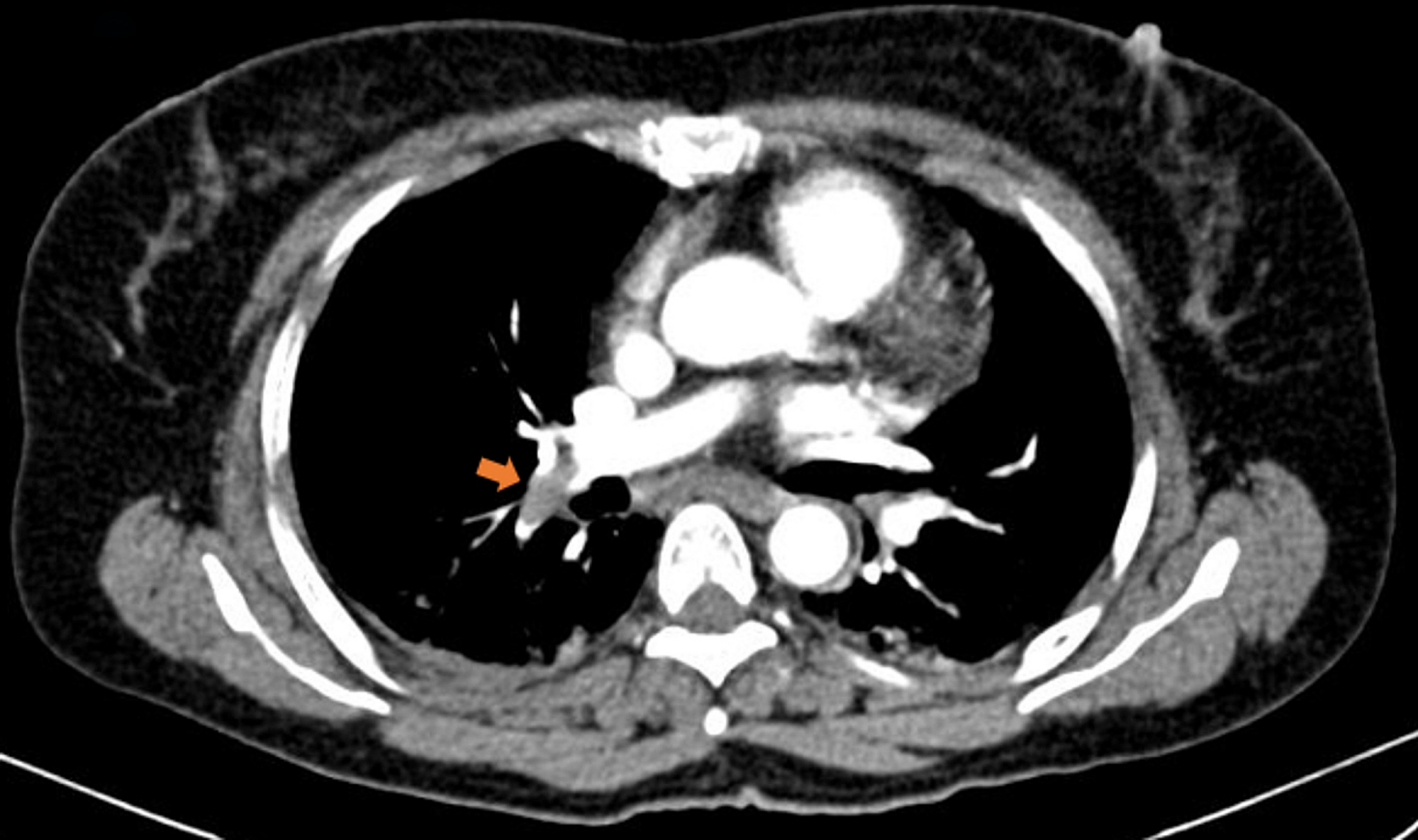
Axial-section image of the contrast-enhanced computed tomography (CECT) of thorax showing a hypodense filling defect (arrow) in the inferior branch of right pulmonary artery suggestive of acute pulmonary thromboembolism.

**Figure 2:**
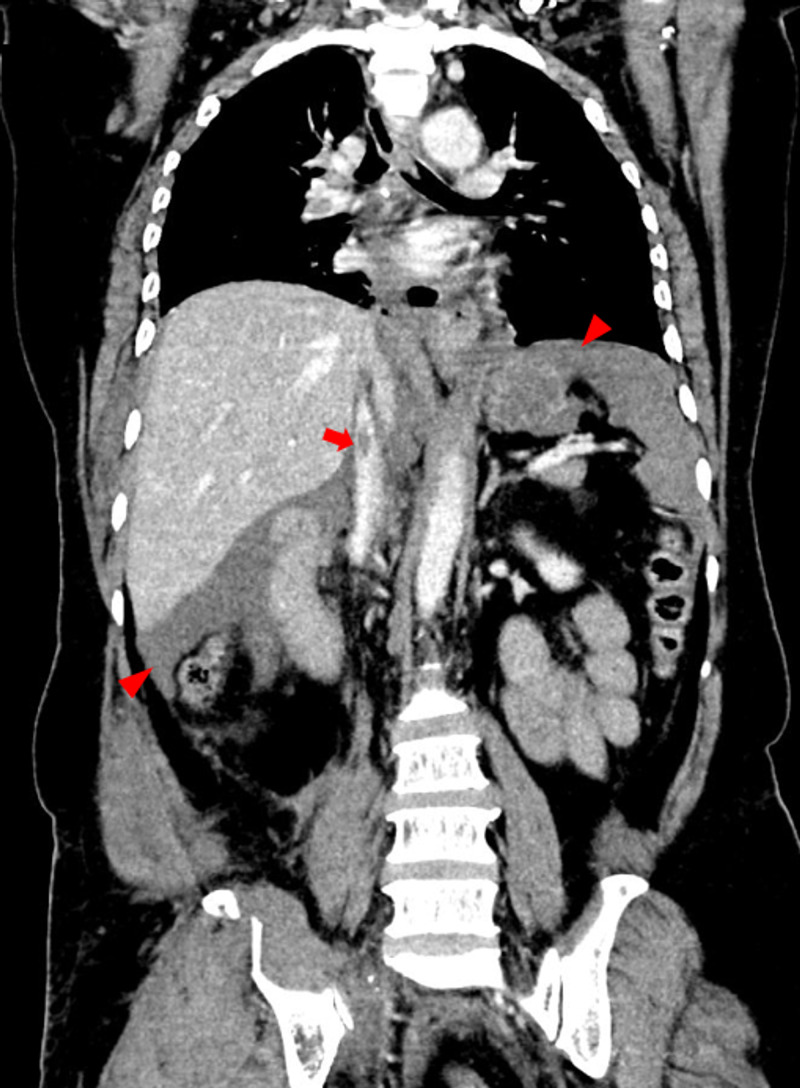
Coronal image of the contrast-enhanced computed tomography (CECT) of thorax, abdomen and pelvis showing a partial thrombus in the infra-hepatic inferior vena cava (arrow). Mild fluid collection seen in the perihepatic and perisplenic spaces (arrowheads) indicating presence of hemoperitoneum.

**Figure 3:**
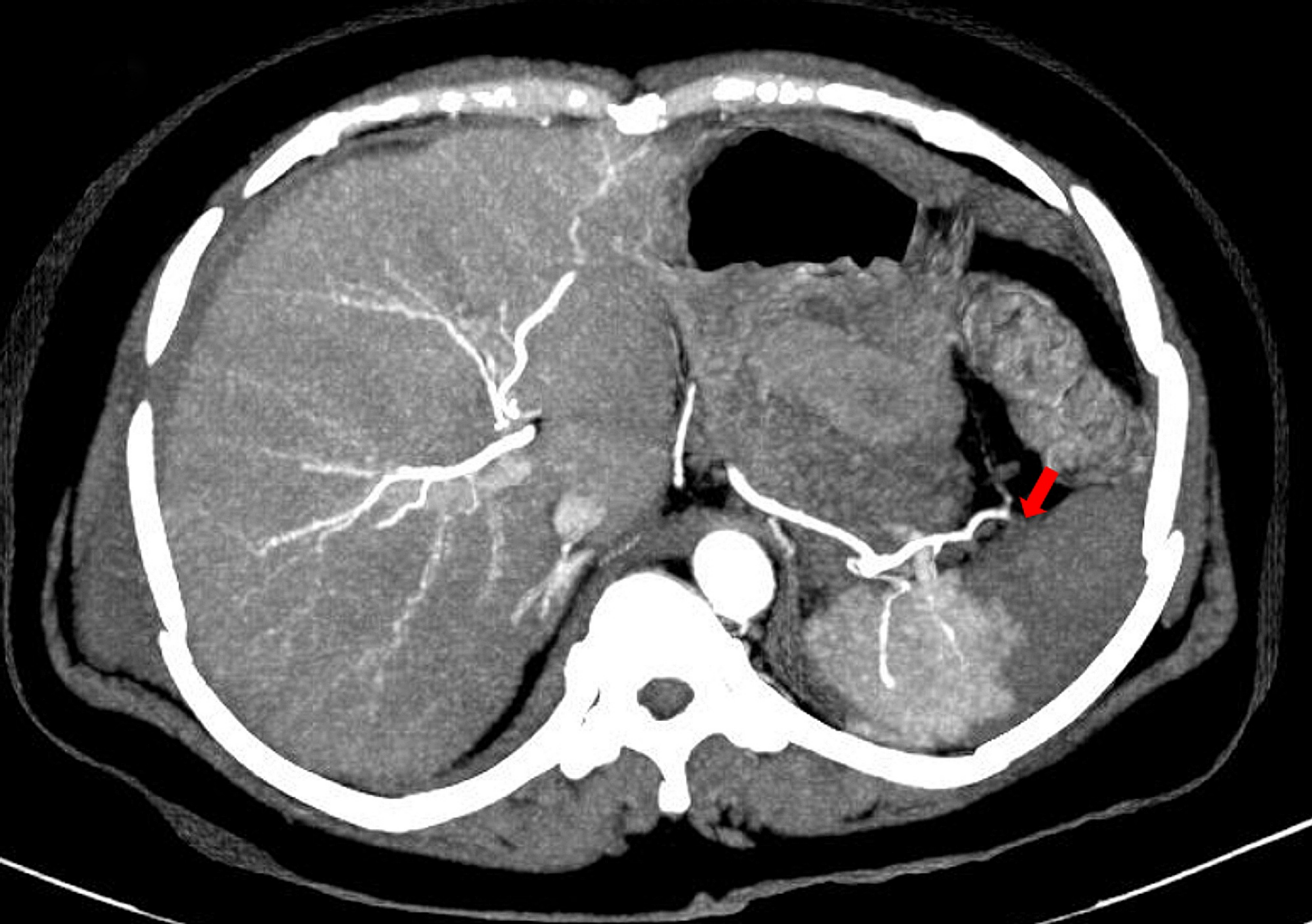
Axial maximum intensity projection (MIP) image of the abdomen showing abrupt termination of the distal branches of the splenic artery (arrow) supplying the infarcted area most probably indicating an embolic occlusion.

A 42-year-old female presented to the casualty with road traffic injuries. She was crossing the road when a tractor hit her. She arrived six hours after the injury in a hemodynamically stable condition with Glasgow Coma Score (GCS) of 15. She was complaining of lower abdominal pain and there were multiple bruises and abrasions on her torso and thighs. The lower abdomen was tender on palpation. Bedside focused assessment with sonography in trauma (FAST) was performed and free fluid was noted in the perisplenic and perihepatic windows. A computed tomography (CT) scan of chest, abdomen, and pelvis was performed. There was evidence of pulmonary embolism in the right pulmonary artery (Figure 1). A thrombus was seen in the inferior vena cava (IVC) and there was mild hemoperitoneum in the perihepatic and perisplenic regions (Figure 2). A wedge-shaped infarct was noted in the spleen (Figure 3). Pelvic bones, right 7th rib, and transverse process of L1 vertebra were fractured. The pelvis was fractured at multiple places in a comminuted fashion.

A provisional diagnosis of pelvic fracture with pulmonary embolism and splenic infarction was made. Departments of pulmonary medicine, cardiology, and orthopedics were consulted. Electrocardiogram (ECG), troponin-T, 2-dimensional echocardiogram (2D ECHO), retinal examination, and D-dimer assays were performed. In ECG, the ST segment was elevated and the test for troponin-T was positive. Other tests were normal. Conservative treatment with injection enoxaparin, injection ceftriaxone, and pain killers was started. Vitals and oxygen saturation were monitored continuously and we examined the abdomen every eight hourly during the first 24 hours. We enquired about the symptoms and performed palpation, auscultation, and abdominal girth measurement at these times. The frequency of examination was reduced to twice daily after the first day as the vitals and the hematocrit were stable. During the first two days, there was mild to moderate continuous lower abdominal pain but it was soft on palpation.

**Figure 4:**
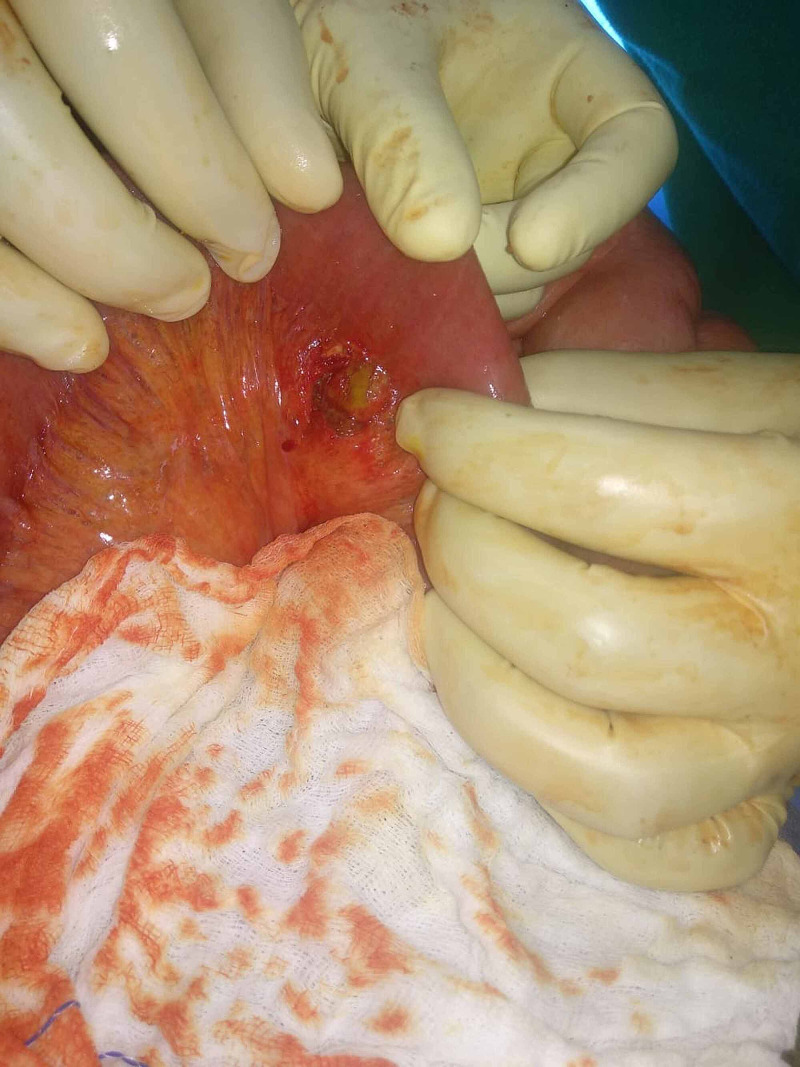
Intraoperative photograph showing mesenteric tear.

On day three, the pain became colicky and was spread all over the abdomen. She was not passing flatus or motion since one and a half days. The pulse rate was 112/min and the blood pressure 98/68 mmHg. The abdomen was distended and was tender on deep palpation. The bowel sounds were exaggerated. On digital rectal examination, the rectum was empty. In view of deteriorating symptoms and possibility of intestinal obstruction, an exploratory laparotomy was performed. There was a small contusion a small mesenteric tear in the ileum around 100 cm proximal to the ileocecal junction (Figure 4). The spleen was normal except for a small capsular tear. A double-barrel ileostomy was made after resecting the contused bowel segment.

The post-operative period was uneventful and the patient was shifted to orthopedics department for further management.

## Discussion

The splenic artery is an end artery and its occlusion results in splenic infarction. The infarct usually resolves spontaneously without any complication. Miller et al. studied 13 patients with traumatic splenic Infarction. They followed 11 of them with computed tomography scans. They found that in the majority the spleen recovered to normal within a few days [1].

Pulmonary embolism is a known complication of major trauma. O'Malley et al. studied 1316 patients of trauma who survived for more than one day and found evidence of pulmonary embolism in 30 of them. Pelvic fracture, age above 55 years, and severe trauma were major factors responsible [2]. Hypothetically the emboli originating from trauma can also block the splenic artery leading to simultaneous occurrence of pulmonary embolism and splenic infarction. However, such an incidence has never been described following trauma. Sugarman et al. described this complication in a non-trauma patient of sickle-cell trait. The patient developed a pulmonary embolism and the resultant hypoxemia caused splenic infarction [3].

The risk of early death in pulmonary embolism is categorized into high-risk and non-high-risk. This is based on three markers - clinical markers, right ventricular dysfunction markers, and myocardial injury markers. The non-high-risk cases can be managed with anticoagulants only [4]. The troponin-T was raised in our case and she belonged to intermediate-risk in the non-high-risk category. Therefore, treatment with injection enoxaparin was started.

Evaluation of the abdomen in trauma is complex. Serial clinical examination and computed tomography scan of the abdomen are useful in hemodynamically stable patients. Serial clinical examination includes physical examination of the abdomen and recording of vitals and hematocrit every eight hourly during the first 24 hours. This is usually not continued after 24 hours in penetrating trauma. However, in blunt trauma the duration of observation is not well defined [5,6]. CT scan of the abdomen is an equally important investigation. It is more useful in patients with blunt trauma and those having poor GCS. It helps to find the source of bleed and also grades the solid organ injuries. It is not a good investigation for detecting intestinal and mesenteric injuries [7,8].

In our case CT scan revealed multiple emboli, hemoperitoneum, and bony injuries. There was evidence of pulmonary embolism, splenic infarction, and embolus in intrahepatic IVC. We started injection enoxaparin and observed the abdomen. We performed exploratory laparotomy because the abdominal pain was not subsiding and CT scan was not able to explain this. An unsuspected intestinal injury was detected. Prolonged serial clinical assessment helped in managing this case. Splenic infarction with pulmonary embolism is a rare and serious complication following trauma.

## Conclusions

Splenic infarction with pulmonary embolism is a rare and serious complication following trauma. Computed tomography scan and serial clinical assessment was helpful in managing this case. We identified most of the injuries with CT scan and a missed intestinal injury was detected on prolonged clinical assessment. Splenic infarct resolved spontaneously and the pulmonary embolism was managed conservatively.

## References

[1] Miller LA, Mirvis SE, Shanmuganathan K, Ohson AS (2004). CT diagnosis of splenic infarction in blunt trauma: imaging features, clinical significance and complications. Clin Radiol.

[2] O’Malley KF, Ross SE (1990). Pulmonary embolism in major trauma patients. J Trauma Acute Care Surg.

[3] Sugarman J, Samuelson WM, Wilkinson RH, Rosse WF (1990). Pulmonary embolism and splenic infarction in a patient with sickle cell trait. Am J Hematol.

[4] Task Force on Pulmonary Embolism European Society of Cardiology (2000). Guidelines on diagnosis and management of acute pulmonary embolism. Eur Heart J.

[5] Cothren Burlew C, Moore EE (2019). Trauma. Schwartz’s Principles of Surgery.

[6] Breigeiron R, Breitenbach TC, Zanini LG, Corso CO (2017). Comparison between isolated serial clinical examination and computed tomography for stab wounds in the anterior abdominal wall. Rev Col Bras Cir.

[7] Ochsner MG, Knudson MM, Pachter HL (2000). Significance of minimal or no intraperitoneal fluid visible on CT scan associated with blunt liver and splenic injuries: a multicenter analysis. J Trauma.

[8] Frick EJ, Pasquale MD, Cipolle MD (1999). Small-bowel and mesentery injuries in blunt trauma. J Trauma Acute Care Surg.

